# Supplementary material for: SARS-CoV-2 Symptoms during the Omicron Surge Differ between Boosted and Vaccinated Non-Boosted Persons
Source: Vaccines (Basel). 2024 Mar 19;12(3):327. doi: 10.3390/vaccines12030327 (PMC10975987; doi:10.3390/vaccines12030327)

Supplemental Table S1. Characteristics of individuals and symptoms reported according to vaccination status with boosted defined as booster receipt more than 14 days before infection.

		Not Boosted	Boosted	p value
		N= 120	N= 273	
**Gender = Female**, No. (%)		72 (60.0)	155 (56.8)	0.581
**Age in years,** Mean (SD)*		31.2 (10.4)	31.5 (12.3)	0.816
**Student versus Employee Status,** No (%)				0.143
Student		80 (66.7)	203 (74.4)	
Employee		40 (33.3)	70 (25.6)	
**Days Since Last Vaccine Until Infection,** Mean (SD)		221.2 (97.1)	128.8 (60.9)	<0.001
**Primary Symptoms Present, No. (%)**				
Cough		96 (80.0)	231 (84.6)	0.305
Fever- present or temperature $\geq$ 100 ^{0}F		63 (52.5)	148 (54.2)	0.826
Nasal congestion		53 (44.2)	160 (58.6)	0.009
Sore throat		52 (43.3)	130 (47.6)	0.444
Headache		41 (34.2)	69 (25.3)	0.087
Body/ muscle aches		38 (31.7)	60 (22.0)	0.044
Fatigue		27 (22.5)	73 (26.7)	0.451
Shortness of breath		23 (19.2)	32 (11.7)	0.058
Gastrointestinal symptoms		11 (9.2)	17 (6.2)	0.295
Change in taste or smell		7 (5.8)	6 (2.2)	0.073
Nasal congestion and/or sore throat		78 (65.0)	209 (76.6)	0.019
**Number of Primary Symptoms Reported,** Mean (SD)		3.4 (1.8)	3.4 (1.5)	0.851
Other Symptoms, No (%)				0.468
0		106 (88.3)	234 (85.7)	
1		11 (9.2)	35 (12.8)	
2		3 (2.5)	4 (1.5)	
**Number of Primary Plus Other Symptoms,** Mean (SD)		3.6 (2.0)	3.5 (1.6)	0.927
**Days Symptoms Present,** Mean (SD)		7.0 (3.1)	6.4 (2.9)	0.044
Cases before January 1, required isolation = 10 days	n= 76	7.4 (3.1)	6.1 (2.2)	0.098
Cases on/after January 1, required isolation = 5 days	n=317	6.6 (3.1)	6.3 (2.8)	0. 574
**Days Isolated,** Mean (SD)				
Cases before January 1, required isolation = 10 days	n= 76	11.0 (1.6)	11.0 (1.3)	1.000
Cases on/after January 1, required isolation = 5 days	n=317	10.2 (2.9)	8.4 (2.9)	< 0.001

*SD= Standard deviation

Supplemental Figure S1. The correlation between the date of infection represented as calendar week on the x-axis and the weeks between receipt of booster vaccination and date of SARS-CoV-2 infection represented on the y-axis.

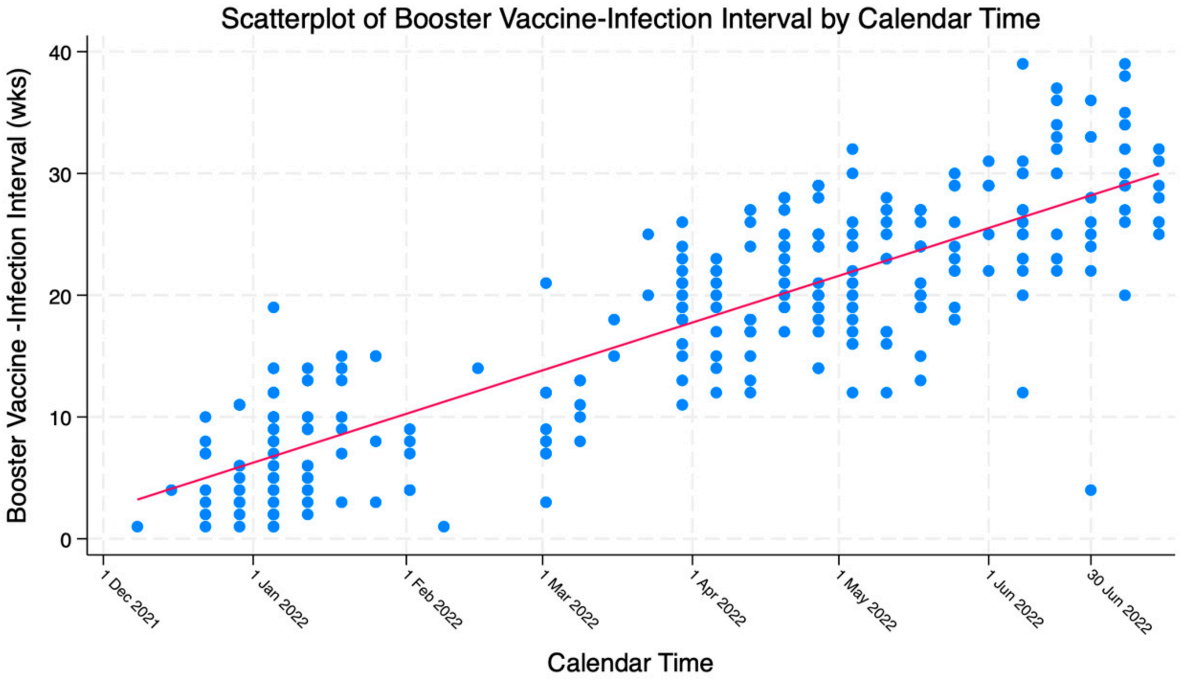

Supplement: Supplementary file 1 [file vaccines-12-00327-s001.zip › vaccines-2874524-supplementary.pdf]
